# The relationship between S1 screw loosening and postoperative outcome in patients with degenerative lumbar scoliosis

**DOI:** 10.1186/s12891-022-05107-0

**Published:** 2022-02-28

**Authors:** Fei Xu, Siyu Zhou, Da Zou, Weishi Li, Zhuoran Sun, Shuai Jiang

**Affiliations:** 1 Orthopaedic Department, Peking University Third Hospital, No. 49 North Garden Road, Haidian District, Beijing, 100191 China; 2 Peking University Health Science Center, No. 38 Xueyuan Road, Haidian District, Beijing, 100191 China; 3 Beijing Key Laboratory of Spinal Disease Reasearch, Beijing, China

**Keywords:** S1 screw loosening, Degenerative lumbar scoliosis, Osteoporosis, Postoperative outcome, Oswestry disability index

## Abstract

**Background:**

When choosing S1 as the lowest level of instrumentation, there are many complications may come out such as S1 screw loosening. Facing this problem, there has been various techniques for the protection of S1 screw including sacropelvic fixation, bicortical or tricortical insertion of S1 screw.

**Objective:**

This study aimed to explore the risk factors for the S1 screw loosening, then to demonstrate the relationship between S1 screw loosening and postoperative outcome for patients with degenerative lumbar scoliosis (DLS).

**Methods:**

Patients who underwent lumbosacral fixation for DLS were evaluated retrospectively. They were divided into two groups according to the S1 pedicle screw at the follow-up. Age, gender, bone mineral density, body mass index, history of smoking, the number of instrumented levels, comorbidities, complications and radiological parameters were collected. We established logistic regression analysis to determine independent risk factors for S1 screw loosening and multiple linear regression to identify whether S1 screw loosening would influence postoperative clinical outcome.

**Results:**

S1 screw loosening rate was up to 41.0% (32/78). Patients were older in the S1 screw loosening group than those in the control group (*P* < 0.05). Compared with the control group, the rate of osteoporosis was higher in screw loosening group than that in the control group (*P* < 0.05). Older age and osteoporosis were independent risk factors for S1 screw loosening (*P* < 0.05). In the screw loosening group, the rate of hypertension was higher than that in the control group (*P* < 0.05). The relationship of S1 screw loosening and ODI was not significant in the multiple linear regression (*P* > 0.05). The clinical outcome was similar in the S1 screw loosening group and control group (*P* > 0.05).

**Conclusion:**

Older age and osteoporosis are independent risk factors for the S1 screw loosening. Patients with complication of S1 screw loosening are not always along with worse clinical outcome. We should consider potential benefit, complications and medical cost when choosing the lowest instrumented vertebrae for patients with DLS.

## Introduction

Recently, some studies have reported that prevalence of adult scoliosis ranged from 8.3 to 68% [1–3]. And when the patients got poor therapeutic effect from conservative treatment, operation became the suitable scheme. As for the selection of the fixed levels, especially for the patients who had disc degeneration, foraminal stenosis, spondylolisthesis and oblique take-off at L5–S1 [4–7], some studies reported that choosing the first sacral vertebra (S1) as the lowest instrumented vertebrae was better. Because L5-S1 segment was more likely to get lumbar disc degeneration than other segments, which usually needed revision surgery [8].

However, lumbosacral fixation bright a high rate of complications including pedicle screw loosening or pseudarthrosis [9–13], whose rate was up to 20–60%, and it was the frequently cited reason for reoperation (25%) [9, 11, 12, 14–17]. The first reason might be that the instrumentation at L5-S1 was under more stress [18]. In addition, S1 pedicle was shorter and had larger diameter than lumbar pedicle, leading to the screw lacking holding power [4, 6, 19].

Thus, different instrumentation and fixation techniques have been proposed, including iliac screws [15]. S2-alar-iliac screw [20] and unilateral pelvic screw fixation [21]. Consequently, iliac screws were effective in protecting S1 screws from screw failure by either breakage, loosening or pullout [15]. Thus, extension of the instrumentation to the pelvis or iliac wings has gained increasing interest.

The risk factors of the S1 screw loosening are still in the dispute. Besides, there still lacks the evidence that inserting the iliac screws simply for preventing S1 screw loosening can contribute to a better clinical outcome for patients. On the other hand, iliac screws require extensive subfascial dissection, increasing the rate of complications such as implant prominence [22], deep infection and poor wound-healing. Meanwhile, several studies [23–25] have shown increased rigidity of lumbosacral fixation techniques contributing to late sacroiliac joint arthritis and pain.

So this study aims to evaluate the risk factors of screw loosening at sacrum and explore the relationship between S1 screw loosening and postoperative clinical outcome in the patients with degenerative lumbar scoliosis (DLS).

## Methods

Patients with degenerative lumbar scoliosis who had undergone instrumentation to the sacrum were evaluated retrospectively. They were divided into two groups according to the status of the pedicle screws at the follow-up X-ray (S1 screw loosening group and S1 screw non-loosening group). Inclusion criteria were as follows: (1) Cobb angle> 10°; (2) Instrumentation to S1; (3) Age ≥ 45 years at the time of surgery; (4) Complete preoperative and postoperative radiographic and functional evaluation data. (5) At least 2 years follow-up. Exclusion criteria: (1) History of idiopathic adult scoliosis; (2) History of ankylosing spondylitis, neuromuscular diseases, fracture; (3) Revision operations because of serious mechanical complications during the follow-up; (4) Patients underwent sacropelvic fixation with additional iliac screws.

We used the posterior midline approach uniformly for all patients. All patients underwent internal pedicle screw fixation and a decompressive laminectomy. And all patients needed interbody fusion received PLIF or TLIF. The lateral extent of decompression was considered complete when the traversing nerve roots were observed. During decompression, the lamina and spinous processes of the fused cranial vertebra were partially retained to preserve the connection between the posterior ligament complex and the neighboring spinous process. The capsule of the cranial facet joint was also protected during the surgery. Following decortication of transverse processes and posterolateral bone, autograft was placed in the posterolateral intertransverse space. An autologous graft with a PEEK cage was obtained from the decompressed lamina and processed. Mobility was restored in all patients within 3 to 5 days after surgery. The patients could exercise the back muscles 3 weeks after surgery and wore a lumbar protective band for 3 months after surgery.

Individual information including age, gender, bone mineral density, history of smoking, menopause, number of instrumented levels, body mass index (BMI), comorbidities (hypertension, diabetes mellitus and coronary artery disease) and complications were collected in these patients. Patients’ intraoperative blood loss, operative time, and hospital stays were reviewed. Preoperative clinical function questionnaire including Oswestry Disability Index (ODI) scores for patients was completed on admission for surgery without assistance. And all enrolled patients were followed-up for at least 2 years from the date of surgery. Questionnaire was completed in the hospital’s outpatient room at the final follow-up.

All the subjects undertook the whole-spine anteroposterior and lateral standing radiograph including their hip joints. Then the following sagittal parameters were measured preoperatively, soon after surgery and at the follow-up period in the PACS system (Picture Archiving and Communication System, USA), including Cobb angle, lumbar lordosis (LL), sagittal vertical axis (SVA), T1 pelvic angle (TPA), pelvic incidence (PI), and pelvic tilt (PT).

The loosening of S1 screws was evaluated by two experienced surgeons. Evidence of a radiolucent zone around the S1 screw was evaluated, and it was determined to be positive when it was more than 1 mm in the thickness region around the screw in the x-ray [26]. The judgement of screw loosening was mainly based on X-ray in this study, which was also the widely-used way to judge the screw loosening [26]. Only if the judgement of screw loosening was not clear in X-ray, we would use the CT scan to help the judgement.

The statistical analysis was performed using SPSS 23.0 software. The independent samples Student’s t-test was used for continuous variables. Chi-squared test was used for categorical data. The significance was defined as *P* < 0.05. Logistic regression analysis was performed to determine which parameter was independently associated with S1 screw loosening. The correlation of ODI and S1 screw loosening was analyzed by multiple linear regression.

## Results

### Demographics

This study included 78 patients (14 males and 64 females), with an average age of 63.0 years (range 45–80 years). The rate of S1 screw loosening was up to 41.0% (32/78). The demographic characteristics were summarized in Table 1.

**Table 1:** Demographic characteristics

	S1 screw non-loosening group	S1 screw loosening group	*P* value
Age	61.5 ± 7.3	65.3 ± 4.7	0.011
Sex (Male/Female)	9/37	5/27	0.656
BMI	26.3 ± 4.3	25.8 ± 4.3	0.67
Number of fused levels	6.2 ± 1.8	6.2 ± 2.0	0.968
Osteoporosis	20.6%(7/34)	75.0%(18/24)	<0.001
L1-L4 average T score	−0.6 ± 2.2	−1.7 ± 1.6	0.034
T score of total hip joint	−1.0 ± 1	−1.6 ± 0.7	0.033
History of smoking	6.5%(3/46)	3.1%(1/32)	0.504
Menopause	78.3%(36/46)	78.1%(25/32)	0.989
Hypertension	32.6%(15/46)	62.5%(20/32)	0.009
Diabetes mellitus	23.9%(11/46)	21.9%(7/32)	0.834
Coronary artery disease	2.2%(1/46)	12.5%(4/32)	0.067
Fusion rate	95.7%(44/46)	90.6%(29/32)	0.373
Interbody fusion	78.3%(36/46)	75%(24/32)	0.789
L5/S1 interbody fusion	19.6%(9/46)	31.3%(10/32)	0.237
Deep infection	2.2%(1/46)	6.3%(2/32)	0.357
Cerebrospinal fluid leakage	2.2%(1/46)	0%(0/32)	0.401

The average age was 65.3 ± 4.7 years in the S1 screw loosening group, which was higher than 61.5 ± 7.3 years in the control group (S1 screw non-loosening group) (*P* < 0.05). The fused levels in the screw loosening group was 6.2 ± 2.0 (mean: 6.2, median:6.0), range from 3 to 12, and the fused levels in control group was 6.2 ± 1.8 (mean:6.2, median:6.0), range from 4 to 12 (*P* > 0.05). There were 60 patients received interbody fusion, and 75% patients (24/32) in S1 screw loosening group received interbody fusion, which was similar with that (78.3%, 36/46) in the control group (*P* = 0.789). Compared with the control group (20.6%, 7/34), patient’s rate of osteoporosis (75.0%, 18/24) was much higher in S1 screw loosening group (*P* < 0.05). Univariate analysis was performed for the effects of comorbidities for S1 screw loosening in the Table 1 (history of smoking, hypertension history, diabetes mellitus history, menopause and coronary artery disease history). The rate of hypertension in the S1 screw loosening group was higher than that in the control group (62.5% vs 32.6%; *P* < 0.05). As for the complications, the rate of infection in the screw loosening group was not significantly higher than that in the control group (2 vs 1; *P* > 0.05). The radiological parameters in the S1 screw loosening group and the control group were shown in the Table 2.

**Table 2:** Relationship of radiological parameters and S1 screw loosening

	S1 screw non-loosening group	S1 screw loosening group	*P* value
Cobb angle (°)			
Preoperative	27.8 ± 13.2	28.7 ± 12.1	0.759
Soon after surgery	11.3 ± 7.3	10.2 ± 5.9	0.460
Change	−16.5 ± 8.4	−18.5 ± 8.7	0.298
Final	11.4 ± 6.3	10.3 ± 5.2	0.392
Sagittal vertical axis (mm)			
Preoperative	43.7 ± 44.4	47.2 ± 51.1	0.745
Soon after surgery	25.0 ± 34.2	14.4 ± 41.0	0.252
Change	−21.2 ± 57.3	−31.8 ± 57.7	0.456
Final	46.7 ± 35	54.2 ± 38.2	0.392
PT (°)			
Preoperative	22.4 ± 11.8	24.1 ± 10.5	0.533
Soon after surgery	17.5 ± 10.3	17.3 ± 8.3	0.923
Change	−4.4 ± 10.1	−6.8 ± 7.7	0.265
Final	21.7 ± 10.2	22.6 ± 9.5	0.696
LL(°)			
Preoperative	26.4 ± 15.0	23.0 ± 15.1	0.334
Soon after surgery	37.7 ± 10.0	36.5 ± 11.0	0.619
Change	11.3 ± 13.4	13.5 ± 13.3	0.482
Final	31.9 ± 10.1	30.1 ± 12.7	0.502
Preoperative PI-LL (°)	20.3 ± 16.4	23.8 ± 17.4	0.372
TPA			
Preoperative	20.2 ± 11.8	22.0 ± 11.5	0.503
Soon after surgery	13.5 ± 8.4	12.6 ± 7.9	0.638
Change	−6.2 ± 9.8	−9.5 ± 8.2	0.145
Final	20.1 ± 10.4	21.3 ± 9.2	0.599
Blood loss (ml)	1244.4 ± 709.9	1294.1 ± 946.5	0.798
Operative time (min)	278.4 ± 55.2	277.7 ± 63.5	0.960
Average hospitalization (days)	13.1 ± 6.8	14.6 ± 9.0	0.425
ODI score	27.2 ± 21.6	31.6 ± 18.9	0.366

### Risk factors for S1 screw loosening

We chose the influential factors (*P* < 0.15) such as age, fused levels, osteoporosis, hypertension, coronary artery disease and changed TPA to determine the relationship between risk factors and the loosening of the S1 screw. And we established a logistic regression model in Table 3. As a result, older age and osteoporosis were independent risk factors for S1 screw loosening (*P* < 0.05).

**Table 3:** Logistic regression analysis of risk factors associated with S1 screw loosening

	Odds ratio	95% CI for OR	*P* value
Age	0.153	1.004–1.351	0.044
Instrumented levels	0.071	0.716–1.608	0.731
Osteoporosis (Yes/No)	2.511	2.513–60.31	0.002
Hypertension	1.511	0.767–26.759	0.095
Coronary artery disease	22.121	<0.001	0.999
Changed TPA (°)	−0.042	0.854–1.077	0.481

### Clinical evaluation

The rate of S1 screw loosening was higher in the patients older than 65 years old than that in the patients who were younger than 65 years old (53.1% vs 46.9%; *P* < 0.05). Patients with postoperative PI-LL ≥ 10 had higher rate of S1 screw loosening than that in the PI-LL < 10 group but there is no significant difference (71.9% vs 28.1%; *P* > 0.05) (Table 4A, B).

**Table 4:** Relationship of age, PI-LL at last follow-up and S1 screw loosening

A			
	Age<65 years old	Age ≥ 65 years old	*P* value
S1 screw loosening rate	46.9%(15/47)	53.1%(17/31)	0.044
**B**			
	**PI-LL < 10 at last follow-up**	**PI-LL ≥ 10 at last follow-up**	**P value**
S1 screw loosening rate	28.1%(9/24)	71.9%(23/54)	0.673

We chose the age, sex, instrumented levels, fusion rate, postoperative SVA [27–29] and S1 screw getting loosening as the risk factors for influencing postoperative satisfaction. And we established a multiple linear regression to identify the relationship between them and ODI (Table 5). Consequently, all the six factors including whether S1 screw getting loosening were not related to the ODI score (*P* > 0.05).

**Table 5:** Multiple linear regression analysis of risk factors associated with ODI score

	B	95% CI for OR	*P* value
Age	0.291	−0.503-1.084	0.467
Sex	9.928	−3.3-23.155	0.139
Instrumented levels	−0.590	−3.461-2.282	0.683
Fusion rate	−8.187	−27.16-10.785	0.392
Postoperative SVA	0.116	−0.019-0.252	0.091
Screw loosening	2.163	−7.74-12.066	0.664

## Discussion

The lumbosacral fixation had a high demand in patients with disc degeneration, foraminal stenosis, spondylolisthesis and oblique take-off at L5–S1 [4–7]. Some studies reported that fixation to the sacrum demonstrated better correction of lumbar lordosis than fixation stopping at L5. Besides, fixed to S1 could prevent subsequent development of pre-existed L5–S1 disc degeneration [4–6].

The S1 screw loosening was reported to be about 15.6–54% in patients with lumbar surgeries [30–32]. But there were few studies focusing on the patients with DLS [4]. In our study, the rate of S1 screw loosening was as high as 41.0% (32/78) in the patients with DLS. Schwab et al. and Kim et al. [4, 31] reported that screw loosening was related to age, and in our study, we found that older age was an independent risk factor for S1 screw loosening. Besides, the patients older than 65 years had higher rate of S1 screw loosening than the patients younger than 65 years old. This study showed that there was no significant difference in gender and BMI between two groups, which was consistent with Kim’s study [31]. In our study, patients with hypertension were more in S1 screw loosening group than that in the control group (62.5% vs 32.6%, *P* < 0.05). But in the previous study, there was no significant difference in the rate of hypertension between screw loosening group and the control group (S1 screw non-loosening group) [33]. The reason for the higher rates of hypertension in screw loosening group might be that the patients with hypertension were more likely to have bad lifestyles such as history of smoking and drinking, which were risk factors for screw loosening [34]. In other words, hypertension might be the intermediate factor. Besides, patients with hypertension were associated with poor vascular condition, which might be related with the screw loosening. The fusion rate in the control group was higher, which was similar with Galbusera’s study [35]. Besides, previous studies [36–38] thought osteoporosis was an independent risk factor for S1 screw loosening, which was in agreement with our results. It has been demonstrated that screw loosening was caused by a cyclic cephalocaudad toggling motion of the screw in the bone–screw interface when an axial compression load was transmitted through the plate or rod to the screw [39]. Meanwhile, osteoporotic bone had a markedly lower capability to sustain stress [40]. In this way, for the DLS patients with osteoporosis, the potential S1 screw loosening risk after surgery should be noticed.

To deal with S1 screw loosening after long fusion, some studies [20, 41] reported that solitary fixation should be extended to lower level, such as iliac screws and S2 iliac screws. Iliac screws could be inserted solely into the iliac or through the iliac crest into the sacrum [41]. It has been proven that iliac screws were effective in protecting S1 screws from screw failure by either breakage, loosening or pullout [15, 24]. As for the comparison of S2 iliac screws and iliac screw, S2 iliac screws might be better once a more extensive dissection was not required. The reasons might be listed as followed. First, the S2 iliac screws could enhanced the pull-out strength of the screws, which were biomechanically similar with the iliac screws [42]. Second, the rod linking to the bony anchors was more direct by the S2 iliac screw. Third, the S2 iliac screw could reduce the rate of symptomatic screw prominence [43]. Forth, it could minimize infection rate with less damage of soft tissue. Fifth, the S2 iliac screw made the rod distance become short, which reduced the risk of rod fracture. Therefore, Shen et al. thought that sacropelvic fixation should be considered in any patient with a long construct ending in the sacrum where the proximal construct was at L2 or cephalic [27].

However, according to our results, the clinical outcome of patients with S1 screw loosening and the control group were not significantly different (31.6 vs 27.2, *P* > 0.05), and the difference was also smaller than minimal clinically important difference (MCID) [44]. This finding indicated that S1 screw loosening in patients with DLS might not influence the postoperative health-related quality of life (HRQOL) that much. And in this way, extending the fixation to lower level to protect the S1 screw from loosening might not greatly contribute to the postoperative clinical outcome. Meanwhile, as for disadvantages of fixation to iliac, iliac screws often required an offset connector which could be failure at the end of the construct and the hardware might also lead to skin necrosis [27, 45, 46]. Other common complications included infection (reported rate was around 4% [47]) and loosening of pelvic fixation.

Historically, iliac screws have been mostly used to augment S1 pedicle screws. And in this study, S1 screw loosening might not greatly influence the postoperative outcome for patients with DLS (*P* > 0.05) according to the multiple linear regression with average 31.0 months follow-up. Improving the patients’ quality of life was the fundamental purpose of surgery, and the surgical strategy should center on this concept. Therefore, spinal surgeons needed to consider more factors when choosing the lowest instrumented vertebrae for patients with DLS and pay more attention to patients’ quality of life rather than just focusing on complications.

There were several limitations to our study. Firstly, this study only focused on patients with DLS, so it should be careful when applying these findings in other patients. Secondly, this was a retrospective study without longer follow-up, and screw loosening might not affect patients in the short time, but the influence was unknown in a long term. Thirdly, this study did not include patients with fixation to iliac. In the future, more prospective studies recruiting patients who underwent lumbosacral and lower fixation with longer follow-up should be conducted, to further evaluate the influence of S1 screw loosening and benefit of extending fixation on patient’s clinical outcome.

## Conclusions

S1 screw loosening is a result of multiple factors in patients with degenerative lumbar scoliosis after surgery. Older age and osteoporosis are independent risk factors of S1 screw loosening. Besides, the surgeons are supposed to balance the potential benefit and cost of extending the fixed level when choosing the lowest instrumented vertebrae.

## Data Availability

The datasets used and analysed during the current study are available from the corresponding author on reasonable request.
